# Preferential associations in an unstable social network: applying social network analysis to a dynamic sow herd

**DOI:** 10.3389/fvets.2023.1166632

**Published:** 2023-06-01

**Authors:** Sarah L. Jowett, Zoe Elizabeth Barker, Jonathan R. Amory

**Affiliations:** ^1^Department of Animal Science, Writtle University College, Chelmsford, Essex, United Kingdom; ^2^Department of Animal Behaviour and Welfare, Institute of Genetics and Animal Biotechnology of the Polish Academy of Sciences, Magdalenka, Poland; ^3^Department of Animal Sciences, University of Reading, Reading, United Kingdom

**Keywords:** preferential association, network, connectedness, motivation, pig, social tie

## Abstract

Preferential associations are fitness-enhancing ties between individuals, documented in a range of taxa. Despite this, research into preferential associations remains underrepresented in commercial species, particularly pigs. This study investigates the development of preferential associations in a dynamic sow herd. Preferential associations were defined as approaching a resting sow and then sitting or lying with physical contact with the selected sow, separated by < 1 m from the head or directly next to her, with interaction tolerated for > 60 s. For individual identification, each sow was marked with colored dots, stripes, or both, corresponding to their ear-tag number. Preferential associations were measured over one production cycle of 21 days. Behavioral observations took place on 7 days of the study, with 3 h of behavior per day recorded during peak activity times (08:00–09:00, 15:00–16:00, 20:00–21:00 h). Behaviors were recorded using five cameras, each positioned within the barn to provide coverage of the functional areas. The network metrics applied included in-degree centrality (received ties), out-degree centrality (initiated ties), centralization (the extent to which an individual is central within the network), clustering coefficient (a measure of tie strength), and the E-I Index (a measure of assortment by trait: parity, familiarity, and sociality). Individuals were added and removed during the study, so the centrality metrics of missing sows were weighted. To describe the structure of the network, brokerage typologies were applied. Brokerage typologies include five positions, including coordinators, gatekeepers, representatives, consultants, and liaisons. The results revealed social discrimination in assortment by connectedness even when ties were not reciprocal, and the most connected sows were significantly more likely to be approached than less connected individuals. The most connected sows had significantly higher in-degree and out-degree centrality. With the application of brokerage typologies, the results showed a relationship between connectedness and brokering type, with the most connected sows predominantly engaging in coordinating behavior. The results suggest that the motivation for discrimination in the unstable preferential association network was not founded upon bidirectional interactions. These findings highlight the complexities involved when forming social preferences and present a platform for further exploring the motivations for preferential associations among intensively farmed pigs.

## Research highlights

- Reciprocity is not a motivator for establishing preferential associations.
- There is a relationship between social discrimination and connectedness.
- Brokerage typologies exist in the preferential association network of dynamic sows.
- Individual sociality drives specific brokering behaviors.

## 1. Introduction

Social ties are important relationships that enable the formation of beneficial, complex interactions between gregarious conspecifics. Positive social relationships are ties that bind individuals in different social contexts, such as pair bonds, maternal–offspring bonds, littermate bonds, and peer–peer bonds. These bonds are positive because they provide both physical and psychological fitness-enhancing benefits that support overall wellbeing. In commercial species, these ties are beneficial because they are shown to attenuate social and environmental stressors, support immune system functions, and improve welfare states (1, 2). The development of positive bonds is dependent on the discriminatory social selection of a conspecific, referred to as a preferential association (3). Unlike social support or buffering, which may be context-dependent, non-specific, and transitory (4), preferential associations are considered stable, non-random relationships (5) that extend beyond parent–offspring interactions. Measures of preferential associations cover an extensive range of species-specific behaviors, including tactile contact, play interaction, or proximity-based [i.e., (6–8)].

At the individual level, socially bonded animals experience greater wellbeing, as demonstrated by the relationship between the formation of preferential associations and the positive effects these bonds have on longevity, yield, reproductive success, and offspring survival (1, 5, 9, 10). To date, research has focused on the fitness benefits of preferential associations in wild taxa [e.g., (5, 11–13)]. Individual differences play a key role in the ecological processes underlying social discrimination in wild species, where the phenotypic and genotypic specific traits (i.e., size and sex) of a conspecific can enhance fitness and survival (14). The biological and social benefits acquired from conspecifics can include improved reproduction and offspring survival through mate selection, the learning of skills that increase mating success, predator avoidance, communication skills, and the acquisition of knowledge pertaining to feeding sites (15–18). However, behavioral traits and familiarity have also been shown to drive preferred partner selection (19, 20). Recently, socially selected ties have been observed in studies of commercial species, especially in variable social and physical environments [e.g., goats (21); pigs (7); horses (22); dairy cattle (23, 24); sheep (25)]. Although commercial species face different challenges than their wild counterparts, establishing preferential associations in domestic animals is a step toward understanding how positive interactions can be incorporated into management strategies to mitigate the stressors experienced by intensively farmed species.

The benefits of social bonds in wild boar (*Sus scrofa*) can be characterized by the sustained reciprocal affiliations that develop between mothers and offspring, and between non-related individuals (26, 27) and the resulting cooperative behavior for foraging, caregiving, and predator avoidance (26, 28). Although matriarchal groups are typically stable (29), they are subject to seasonal changes in the social configuration determined by environmental factors (30, 31). Wild boar social behavior is extremely robust when subjected to changes in social configuration. Bieber et al. (27) found that when wild, unrelated, female boars of the same age were housed together, they formed stable groups without the presence of a linear hierarchy. In contrast, in a commercial setting, social structure is degraded due to large herds and dynamic practices (32, 33), where unfamiliar individuals are routinely added and removed from the herd, altering the social configuration. Therefore, establishing long-term preferential associations representative of wild boar relationships presents a challenge for commercial pigs housed in socially unstable networks, to which they are not genetically predisposed (34).

Under seminatural and free-range conditions, piglets form social bonds with non-littermates (35, 36). More recently, Goumon et al. (7) revealed the development of dyadic preferences in a stable group of 12-week-old commercial pigs housed in a mixed herd of three litters, providing further support that social stability enables the development of preferential relationships in early life. Related studies also show that in the absence of social mixing, there is a reduction in socio-negative behaviors (37), which represents a persisting welfare issue in commercial pigs (38). These findings present an opportunity to explore the development of preferential ties at alternative production stages and under different management practices.

Limited research on the development of positive social relationships in dynamic pigs indicates that when social remixing is implemented, the development of stable dyadic bonds remains unresolved (3) due to the confounding factor of preferred lying location (39, 40). Dynamic pigs refer to animals housed in a production system where individuals are routinely added and removed from the social group. A recent spatial proximity study of gestating sows revealed the formation of stable subgroups within a large dynamic herd (41), supporting an attempt at self-regulation of herd size consistent with wild boar behavior. Nonetheless, the study described the community structure of an undirected network. If the assumption of preferential associations or “friendships” refers to a bidirectional, dyadic tie (3, 42), then directed networks must be employed to decipher the true nature of the social bond at the individual level, a perspective shared by authors in their previous studies [i.e., (6, 43)].

The current study extends the understanding of the complex behavioral processes and motivations for the emergence of social bonds in an unstable setting using several social network analysis metrics. The application of social network analysis allows for the assessment of group structure and individual-level behaviors. Group-level metrics, including centralization, density, reciprocity, the clustering coefficient, and external–internal index reveal details of group cohesion, provide an overview of the strength of social ties that exist within the social network, and the capacity to evaluate assortment based on social and biological attributes. Individual-level metrics, including in-degree centrality (received behavior) and out-degree centrality (initiated behavior), allow for the analysis of individual differences in behavior with respect to specific social mechanisms. In the current study, these social mechanisms are determined by the application of subgroups, including the *k*-cores (connectedness) and brokering typologies. Traditionally, brokering behavior is determined with the application of the social network metric of betweenness centrality, which extends beyond dyadic interactions to identify individuals within a social group that may be more influential than conspecifics in a behavioral or biological transmission network (44–46). In affiliative behavior networks, brokering individuals that broker are important for group fitness as they allow for increased social cohesion [e.g., (47)]. Beyond the traditional measure of betweenness centrality, brokering typologies can be applied to enhance the understanding of individual behavioral transmission at the subgroup level. There are five brokering typologies including coordinator, gatekeeper, representative, liaison, and consultant (48). Each of the brokering typologies transfers behavior within and between subgroups in a specific manner. To date, brokering typologies have not been applied to positive behavior networks in animals.

Our previous social network analysis study investigated preferential associations to identify prominent and influential sows in three preferential association networks (45). The findings revealed subgroups (*k-*cores) with an assortment of connectedness and general instability in behavior. We additionally applied brokering typologies to the agonistic networks of the same study herd (49), identifying a range of brokering behavior within and between the subgroups (*k*-cores). This study of agonistic behaviors revealed a relationship between sociality and individual brokering type. The current study investigates the emergence of social bonds in an unstable setting by applying brokerage typologies to a preferential association network of sows housed under unstable social conditions. Preferential associations were observed to be based on resting proximity, and a directed network was implemented to distinguish between the initiator and recipient of the behavior. Our research also evaluated preferential assortment based on attributes, including parity, familiarity (determined by breed group), and sociality (determined by an individual's level of connectedness). Due to the emergence of subgroups in the agonistic and preferential association networks of a dynamic herd, our overarching hypothesis is that individuals engage in a predominant brokering typology within a preferential association network, determined by their level of connectedness.

## 2. Methods

### 2.1. Animals and housing

This study was conducted at Sturgeon's Farm, Writtle University College, Chelmsford, Essex, United Kingdom, which supports an 80-sow unit. The herd consisted of a commercial cross of Landrace x Large White, with parities ranging from one to six. During gestation, animals had free movement within the dry barn (Supplementary Figure 1) to all functional areas and *ad libitum* access to straw. Functional areas included the straw-bedded area, the passageway, the drinking station, and the electronic sow feeder station. The straw-bedded area measured 20 m × 6.5 m, with additional space in the shared passageway of 17 m × 3 m. Two electronic sow feeders and five nipple drinkers were in the passageway. Stocking densities within the barn were variable, resulting from the dynamic system, allowing for ~2.32 m^2^ per sow. The sows were apportioned rations of Delta Renovo TD sow pellets, with individual quotas metered electronically and determined by body size. Sturgeon's Farm operates a dynamic production system in which small groups (~12 sows) are artificially inseminated and mixed into the herd every third Tuesday, following a continuous cycle of production: farrowing week, breeding week, and weaning week. A production cycle is defined as a 21-day observation period in the dry barn. For individual identification, each sow was marked with colored dots, stripes, or both on their backs using a livestock marker, which corresponded to the ear-tag reference number. The number of sows observed in the study was 78.

### 2.2. Video observation data

Observations of the herd were recorded using five H.265 4MP Eyeball PoE infrared dome cameras (Genie, WIP4EBVS). Each camera was positioned in the barn to access the key functional areas, including the straw-bedded area, the passageway, the isolation pen, the feeders, and the drinking station. The footage was recorded continuously onto an H.265 eight-channel network video recorder (Genie, WNVR185) fitted with a 3TB hard drive. The DVR was housed in a side room of the barn, accessible without disturbing the sows, and connected to a 21.5″ LED Hi-Res VGA, DV1 HDMI CCTV monitor (Genie, LM-215). Behavioral data were collected over one production cycle in November 2017. The hours of observation, 08:00–09:00 h, 15:00–16:00 h, and 20:00–21:00 h, were determined by the prior pilot study, which investigated the optimum times for sow activity. Video observations took place on 7 days of the production cycle, including the day before mixing and the day of mixing. Observations then continued for the 3 consecutive days following the mixing event [as in (50)], the period when the social structure is restabilized (51). The seventh and 14th days after mixing was also selected to allow for temporal changes in behavior. Overall, 21 h of video footage were included. The same researcher (SJ) conducted the observations.

### 2.3. Behavioral measures

Behavioral sampling used an all-occurrences (52) recording method for preferential associations in the production cycle. Preferential associations were defined as the social selection of a resting partner (Table 1). The frequency of interactions was recorded between the initiator and recipient of the preferential association to provide a directed and weighted network (in which the total number of interactions for each sow was included), allowing for the distinction between in-degree centrality and out-degree centrality. In-degree centrality refers to the total number of received preferential associations. Out-degree centrality refers to the total number of initiated preferential associations. The positive outcomes recorded toleration of an initiator >60 s. Due to the use of weighted data, threshold filters were applied to the preferential association network. In line with previous, related studies of preferential associations [e.g., (3, 7, 53, 54)], it is required to apply thresholds to the interactions between an initiator and the same recipient. Thresholds were applied to further ensure that potentially random interactions could be discounted, as random interactions should not be considered a preferential association. In this study, there were three thresholds for interaction: the original network (with no thresholds applied), the mean network, and the 1.5 × mean network (Table 2). This allowed for comparisons in group structure and cohesion between the three networks as the threshold level required to be included in the network increased. The mean network, in which the threshold level was set at ≥2 interactions between an initiator and the same recipient, was selected for further analysis.

**Table 1:** Ethogram of preferential associations.

**Behavior**	**Description**
**Preferential associations**	
Social selection of resting partner	Approaching a resting sow and then sitting or lying with physical contact or resting (asleep or awake) next to the selected sow, separated <1 m from the head of the selected sow and directly next to her. The proximity of the approaching sow was tolerated >60 s.

Adapted from Durrell et al. (3).

**Table 2:** Description and threshold levels of preferential associations for the original network (*n* = 78), the mean network (*n* = 70), and the 1.5 × mean network (*n* = 42).

**Network composition**	**Description of the network**	**Threshold measure applied**
Original	The original and unfiltered network, consisting of all preferential associations, including singular interactions taken over the seven days of observations.	Inclusive of 1–5 interactions between any initiator and the same recipient.
Mean	The mean network consisting of the mean weighted degree of preferential associations taken over the seven days of observations.	Inclusive of 2–5 interactions between any initiator and the same recipient.
1.5 × mean	The 1.5 × mean network consisting of the 1.5 × the mean weighted degree of preferential associations taken over the seven days of observations.	Inclusive of 3–5 interactions between any initiator and the same recipient.

### 2.4. Construction of the social network and social network metrics

#### 2.4.1. Centrality measures: betweenness centrality and degree centrality

Betweenness centrality (55) is an individual-level social network metric that provides a measure of the number of times an individual falls along the pathway between two previously unconnected individuals (56). An individual that is not on any pathway between two others will have a betweenness centrality value of 0.

Degree centrality is an individual-level social network metric and refers to the total number of behavioral interactions that an individual has within a network (56). The network included received ties (in-degree centrality) and initiated ties (out-degree centrality). For example, a sow with an in-degree centrality of 15 was the recipient of 15 counts of a preferential association (not necessarily from the same initiator), and a sow with an out-degree centrality of 12 initiated 12 counts of a preferential association (not necessarily to the same recipient). There were potentially 21 h of video observations per sow. However, due to the dynamic nature of the herd, numerous sows (*n* = 14) were not consistently present during the 21 h of observations. For these missing individuals, the data were weighted to account for the number of hours the sows remained absent from the network, with a coefficient applied to the in-degree and out-degree centrality of these individuals. The coefficient assumes that the rate of individual social interactions is consistent across hours. The study is not looking at changes in behavior over time within the production cycle; therefore, this assumption is justified. The coefficient applied to absent sows is y = n/x,

where

y = weighted value of interactions per hour observed.

n = value of either in-degree or out-degree centrality.

x = number of hours observed.

#### 2.4.2. Centralization

Centralization is a group-level social network metric defined as the extent to which the network is dominated by one individual and how central that individual is in the network compared with others (56). The range of centralization is determined by the variance and equality of individual centrality in a social group (57). Inequality of centrality metrics provides a centralization value closer to one, revealing individuals within the group with a disproportionately higher level of centrality than their conspecifics. Decentralized networks reflect greater equality between individual centrality metrics and present a centralization value closer to zero.

#### 2.4.3. External-internal index

The E-I Index is a measure of group embedding and evaluates the extent to which homophily or heterophily based upon ego-similarity is occurring within a network (56). The E-I Index is reported on a scale from −1 (perfect homophily) to 1 (perfect heterophily). The E-I Index was applied to evaluate the extent to which sows assorted by parity, familiarity (based on breeding group), and sociality (based on *k*-core). Parities ranged from one to six, breeding groups (1–7) were organized based on farrowing data, and coreness value (*k*-cores) ranged from one to four.

#### 2.4.4. Density

Density is a group-level social network metric and refers to the proportion of all possible dyadic ties that are present within a network. In a directed network, the maximum number possible is *n* (*n*−1), where *n* is the total number of nodes (56). Nodes are defined as the individuals that comprise a network (56). Density provides a representation of group cohesion and, in this study, relates to the proportion of preferential associations that are engaged. Density is reported on a scale from 0 to 1, and a low density would be closer to 0, indicating little cohesion between herd members. For example, a density of 0.15 would show that only 15% of all potential ties were present within a network.

#### 2.4.5. Reciprocity

Reciprocity is a group-level social network metric that looks at the extent to which a tie between two nodes is mutual (56). Directed networks have four potential dyadic relationships that can occur: A does not have a relationship with B (null dyad); A interacts with B without reciprocation (asymmetric dyad); B interacts with A without reciprocation (asymmetric dyad); or A and B interact with each other (symmetric dyad). The maximum value for reciprocity is 1 (perfect reciprocity), consisting of only symmetric dyads. The minimum value for reciprocity is 0 (anti-reciprocity), consisting of only asymmetric or null dyads.

#### 2.4.6. Clustering coefficient

The clustering coefficient reports on whether there is transitivity in a network, and as a group measure, it is the mean of the individual clustering coefficients for each node (56). The coefficient evaluates whether, if A and B are connected and B and C are connected, to what extent will A and C be connected. If A, B, and C are connected, there is triadic closure (58). The clustering coefficient is reported on a scale between 0 and 1, with 1 being perfect triadic closure and 0 showing no triadic closure. A low clustering coefficient reflects that ties between individuals within a network are weak and a high clustering coefficient shows that ties are strong. This study implemented a weighted overall graph clustering coefficient, described as the most effective method for measuring transitivity (56).

#### 2.4.7. Quantification of the subgroups

*K*-cores have been applied to the preferential association mean network (*n* = 70) to ascertain subgroups based on connectedness. A *k*-core is a sub-graph in which every individual has degree *k* or more connections with conspecifics within the sub-graph (56) and is implemented for the identification of sub-structures within a network (59). The *k*-core (coreness value) reflects the minimum number of nodes a sow connects with but does not show the weight of the interactions (e.g., degree centrality). For example, a K1 sow (connected to at least one other) may have few connections but a high degree of centrality if they interact with conspecifics multiple times. In contrast, a K4 sow (connected to at least four others) may have a low degree of centrality.

#### 2.4.8. The application of brokerage typologies

The brokerage position (48) is an individual-level social network metric that measures the extent to which an individual lies on the directed path between two previously unconnected individuals. Brokerage typologies (Supplementary Figure 2) consist of five brokerage typologies, namely, coordinators, gatekeepers, representatives, consultants, and liaisons (48, 60). Brokerage typologies are not mutually exclusive, with individuals capable of taking on one or multiple roles independently over time. Brokerage typologies require individuals to be sorted by a specific trait, representing subgroups within a network (48). In this study, due to an assortment by sociality, the trait applied was an individual's coreness value (*k*-core). To investigate behavioral differences between brokerage typologies and the formation of social bonds within and between subgroups in the preferential association network, census data, and normalized relative brokerage scores were applied. Census data refer to the actual frequency with which each brokerage typologies is engaged in each subgroup, providing a count at the network level. Normalized relative brokerage refers to the brokerage raw scores divided by the expected value, providing a brokerage profile for each sow, indicating which brokerage typologies they are predominantly engaged in. The normalized relative brokerage profiles determined the brokerage typology to which an individual was assigned.

### 2.5. Social network analysis and statistics

Social network properties for in-degree centrality, out-degree centrality, density, centralization, the E-I Index, reciprocity, the clustering coefficient, *k-*cores, census data, and relative normalized brokerage were performed for the preferential association network in Ucinet 6, version 6.634 (61). To determine the effect of subgroup (*k*-core) and the formation of social bonds (based on preferential associations), data were fitted with generalized linear mixed models (GLMMs) using a negative binomial distribution. GLMMs were performed in R.3.4.1 (62) using the R package lme4, version 1.1-21 (63). For each model, fixed effects were the subgroup (K1, K2, K3, and K4), and random effects were the sow identification number.

### 2.6. Ethics

The study was approved by the Writtle University Ethics Committee on 04/04/17. Form AW1-Animal Welfare, reference: 98363980.

## 3. Results

### 3.1. Visualization of the networks

**Figure 1:**
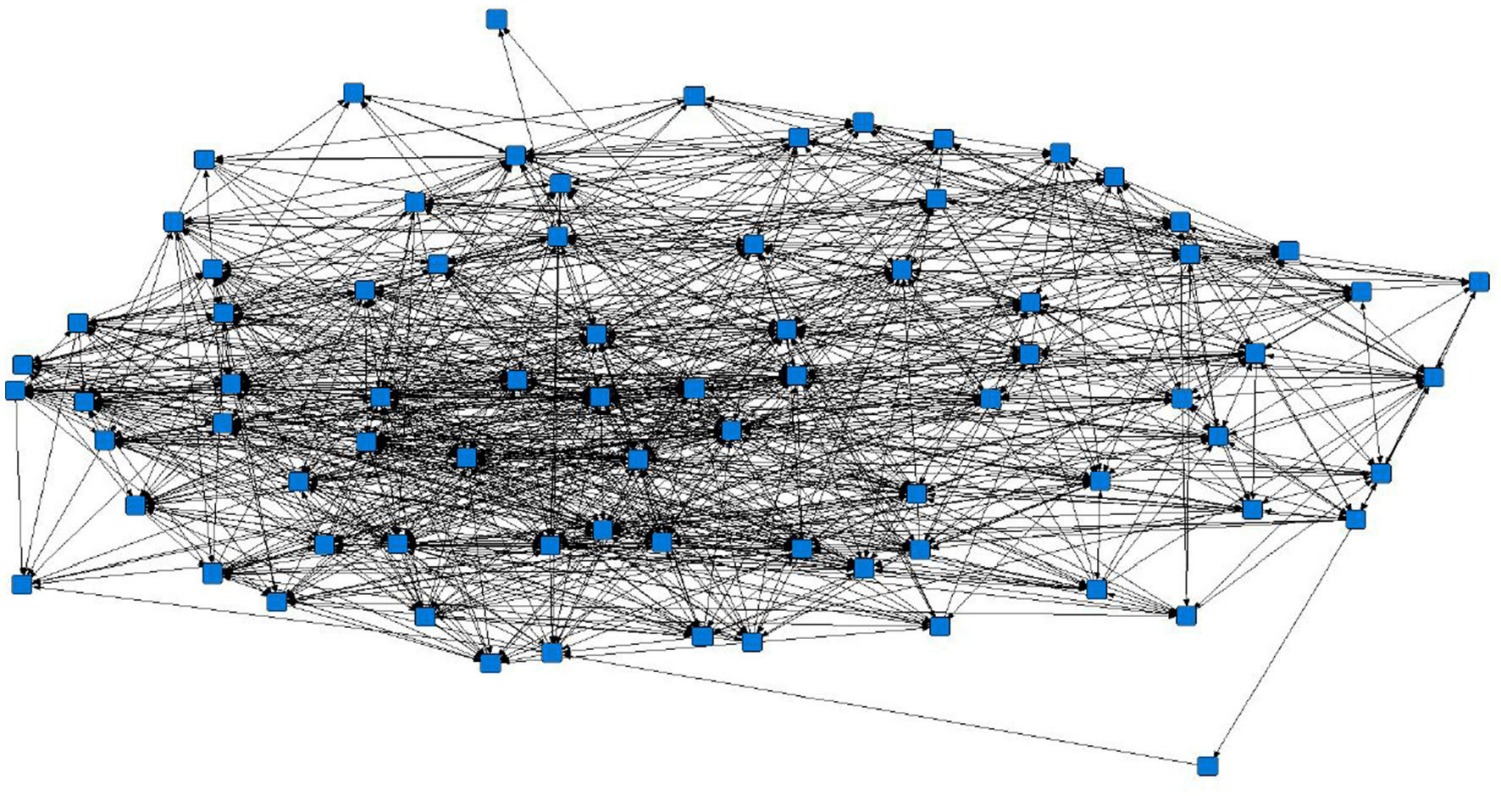
Directed sociogram of all preferential associations in the original network (*n* = 78), observed over a production cycle (21 days) between dry-housed, gestating sows at Sturgeon's Farm, Writtle University College, Essex, UK. The blue squares represent the individual nodes within the network. Previously published in Jowett and Amory (45). Copyrights were obtained.

**Figure 2:**
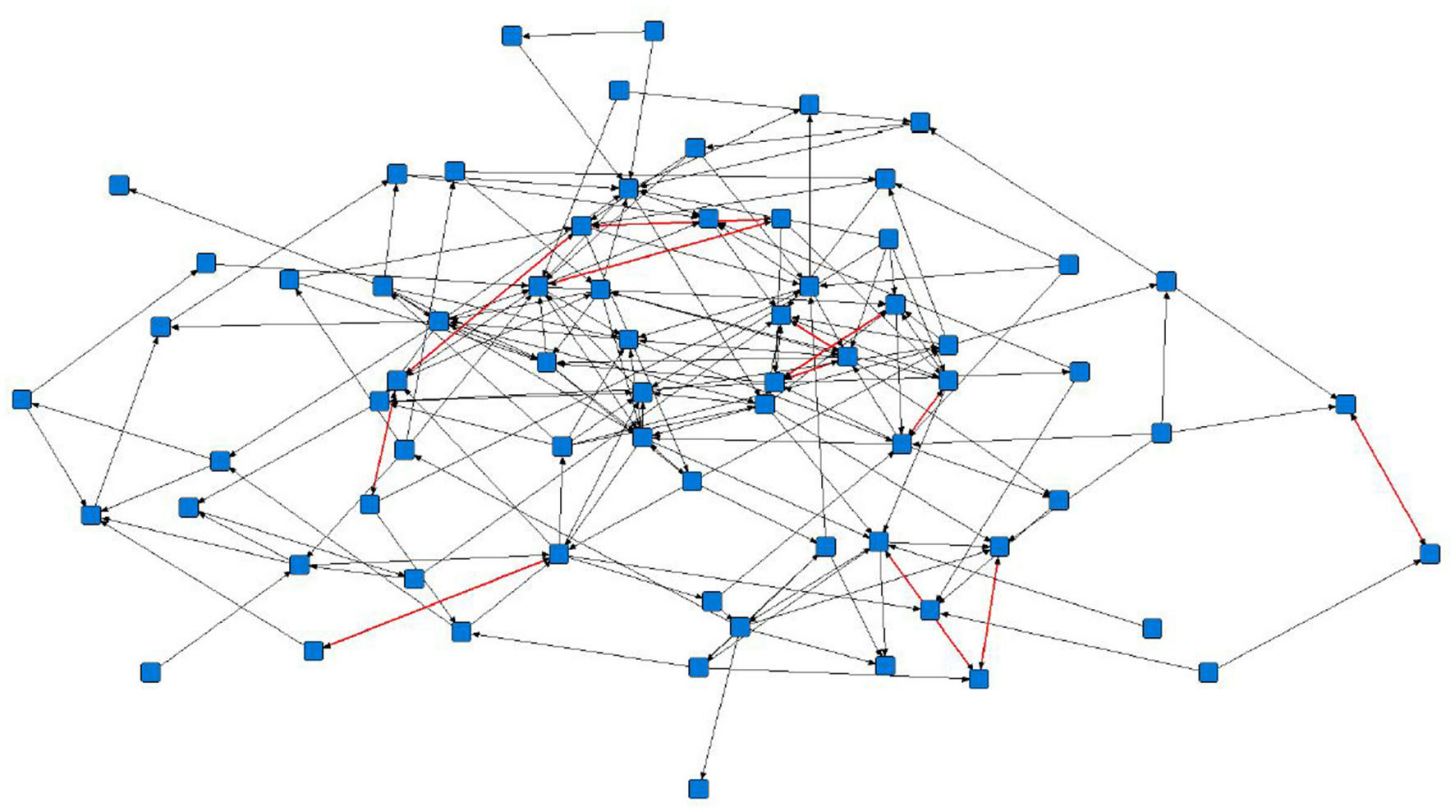
Directed sociogram of all preferential associations in the mean network (*n* = 70), observed over a production cycle (21 days) between dry-housed, gestating sows at Sturgeon's Farm, Writtle University College, Essex, UK. Reciprocated ties are shown as red edges. The blue squares represent the individual nodes within the network. Previously published in Jowett and Amory (45). Copyrights were obtained.

**Figure 3:**
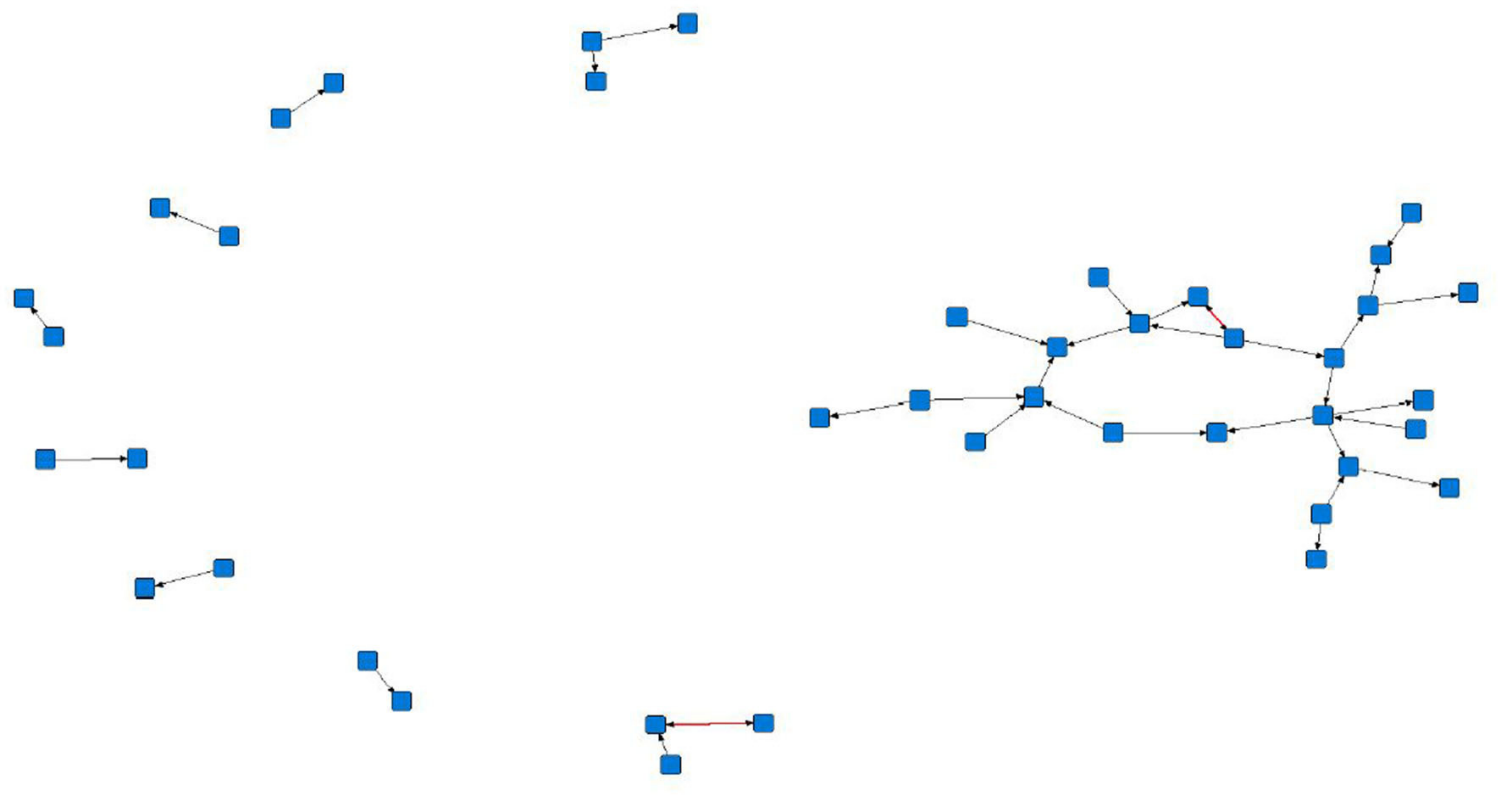
Directed sociogram of all preferential associations in the 1.5 x mean network (*n* = 42), observed over a production cycle (21 days) between dry-housed, gestating sows at Sturgeon's Farm, Writtle University College, Essex, UK. Reciprocated ties are shown as red edges. The blue squares represent the individual nodes within the network. Previously published in Jowett and Amory (45). Copyrights were obtained.

The sociograms illustrated the preferential associations between sows in the unfiltered (Figure 1, *n* = 78), mean (Figure 2, *n* = 70), and 1.5 × mean (Figure 3, *n* = 42) networks. The sociograms showed the total number of interactions across the seven preselected days of video observation, with 21 h of footage. The edges between nodes were weighted and directed. Directed interactions illustrated who approached whom where there was at least one interaction in which proximity was tolerated beyond 60 s. The sociograms highlighted that the cohesiveness of the networks declined as the required threshold of interaction necessary to be included in a specific network increased. Reduced cohesiveness was demonstrated by the increased count of components within each network, with the unfiltered network showing the maximum global cohesion. Reciprocated ties within the sociograms were represented by red lines.

### 3.2. Network descriptive measures and structure

Density revealed that a low proportion of all potential preferential associations were present in all three networks, with only 4 and 2% of possible connections made in the filtered networks (Table 3). Within the 1.5 × mean network, the low proportion of preferential links was consistent with the number of isolates and nodes unconnected to any other node, and the pattern of behaviors revealed a marked increase in the number of isolates as the threshold level increased, with no isolates in the unfiltered network, eight isolates in the mean network and 36 isolates in the 1.5 × mean network. The results indicate a lack of propensity to establish more sustained associations as the threshold for interaction increased and are further supported by the mean degree results. The mean degree for each network decreased as the threshold for interaction increased (Table 3). Overall population centralization (Table 3) for all three networks was low (original = 0.14, mean = 0.13, 1.5 × mean = 0.09), indicating decentralized networks. Comparisons to the mean betweenness centrality also demonstrated that the mean network contains more individuals with greater influence than the other two networks.

**Table 3:** General network metrics for the original network (*n* = 78), the mean network (*n* = 70), and the 1.5 × mean network (*n* = 42).

	**Original network**	**Mean network**	**1.5 × mean network**
**General properties**			
Isolates	0	8	36
Components	1	9	45
**Network-level**			
Density	0.17	0.04	0.02
Mean degree	12.91	5.1	0.88
Centralization degree	0.14	0.13	0.09
Centralization In-degree	0.06	0.09	0.05
Centralization Out-degree	0.04	0.05	0.05
Clustering coefficient	0.231	0.077	0.044
Arc reciprocity	0.264	0.124	0.108
Mean betweenness	73.99	158.37	3.36

The reciprocity of all initiated preferential associations in all three networks did not notably deviate from the density levels (Table 3), suggesting that a high proportion of reciprocated ties were occurring randomly. Nevertheless, as reciprocity values were slightly higher than 0 (original = 0.264, mean = 0.124, 1.5 × mean = 0.108), there was the presence of a small number of mutual connections. Additionally, the clustering coefficients indicated little closure between the triadic interactions (original = 0.231, mean = 0.077, 1.5 × mean = 0.044), and the coefficients hardly differed from density in all three networks (Table 3), suggesting that the preferential ties between any two individuals were weak, with little transitivity occurring.

### 3.3. Application of the E-I Index

The ego-alter similarity of defined attributes within the mean network was measured using the E-I Index (10,000 permutations). No constraints were observed for the given density of group size for each attribute, so the re-scaled E-I Index was not reported. For each attribute, the maximum E-I value was 1 (perfect heterophily/all ties are external), and the minimum E-I value was −1 (perfect homophily/all ties are internal). Parities ranged from one to six, breeding groups (1–7) were organized based on farrowing data, and *k*-cores ranged from K1 to K4 (Supplementary Table 1).

The results show a lack of deviation between the observed E-I Index and the expected E-I Index and positive E-I Index values for the parity and breeding group, revealing that the ego-alter assortment in the mean preferential association network was not motivated by similarities of parity or familiarity through a breeding group. In comparison, the assortment was driven by the *k*-core subgroup, as the negative E-I Index value also deviated more significantly from the expected outcome. Relative comparisons with the standard error suggested a rejection of the null hypothesis, with results appearing to be less random between members of the same *k*-core. This finding was supported by the results of the random permutations test, which show that associations based on *k*-core did not occur by chance (*p* < 0.05).

### 3.4. Application of the subgroups (*k*-cores)

**Figure 4:**
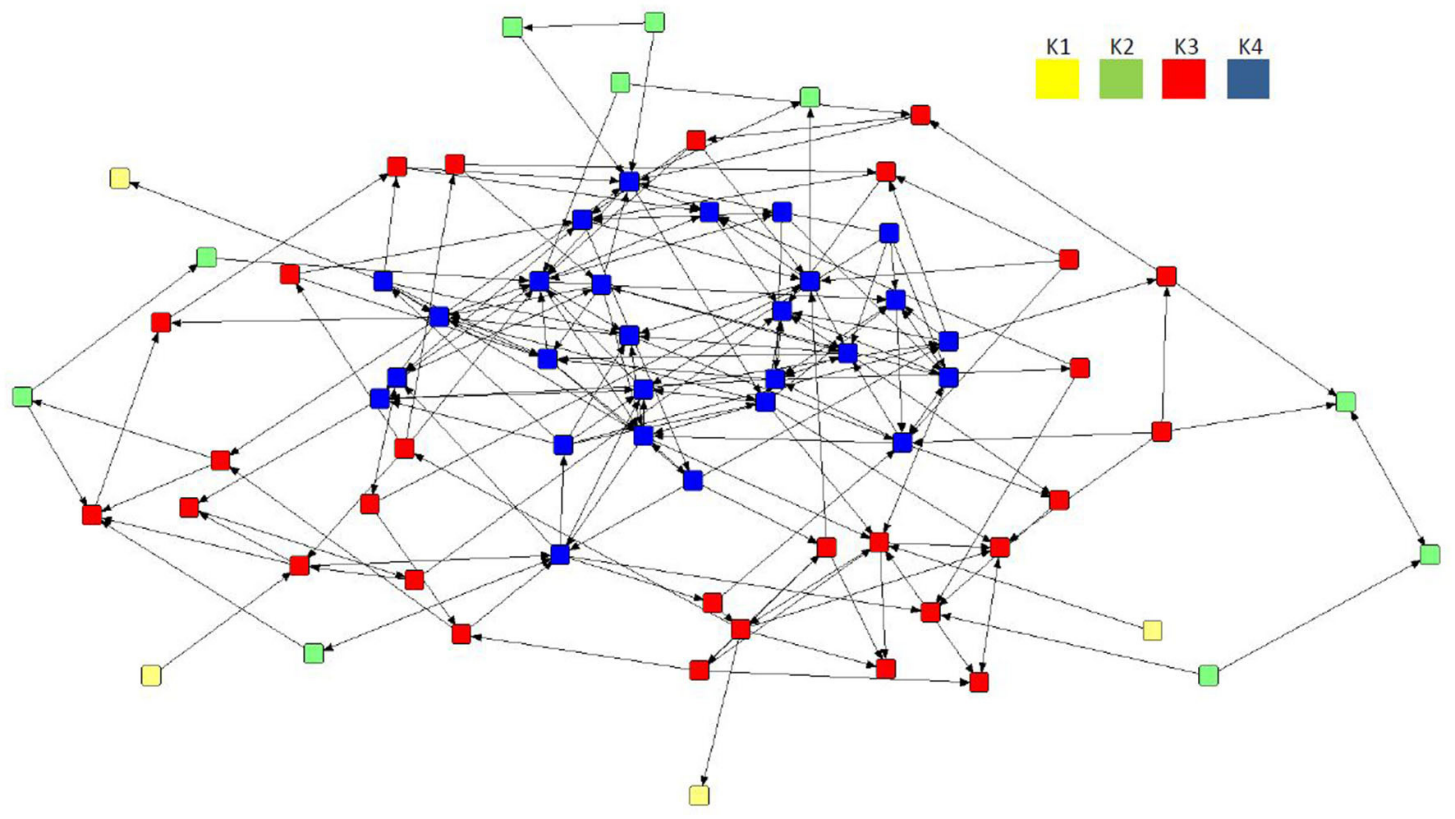
Directed preferential association sociogram of the mean network (*n* = 70) with the *k*-core analysis applied, showing the formation of four subgroups within the herd including K4 (*n* = 27), K3 (*n* = 29), K2 (*n* = 10), and K1 (*n* = 4). The legend denotes the coreness value for each subgroup.

Despite a lack of cohesiveness and clustering demonstrated in all three preferential networks overall (Table 3), the application of the *k*-cores to the mean network revealed the presence of four interconnected core areas (Figure 4): K1 (*n* = 4), K2 (*n* = 10), K3 (*n* = 29), and K4 (*n* = 27). The mean network was selected as it demonstrated the network with the highest mean betweenness (158.37) and centralization in-degree (0.09), indicating the network with potentially more influential sows. The maximal subgroup (K4, *n* = 27) reflected the most cohesive region of the network, although K3 (*n* = 29) represented the largest group of connected individuals. Two further subgroups were located on the periphery of the mean network, albeit consisting of a much smaller number of nodes (K2, *n* = 10; K1, *n* = 4).

### 3.5. Sociability and the subgroups (*k*-cores)

**Figure 5:**
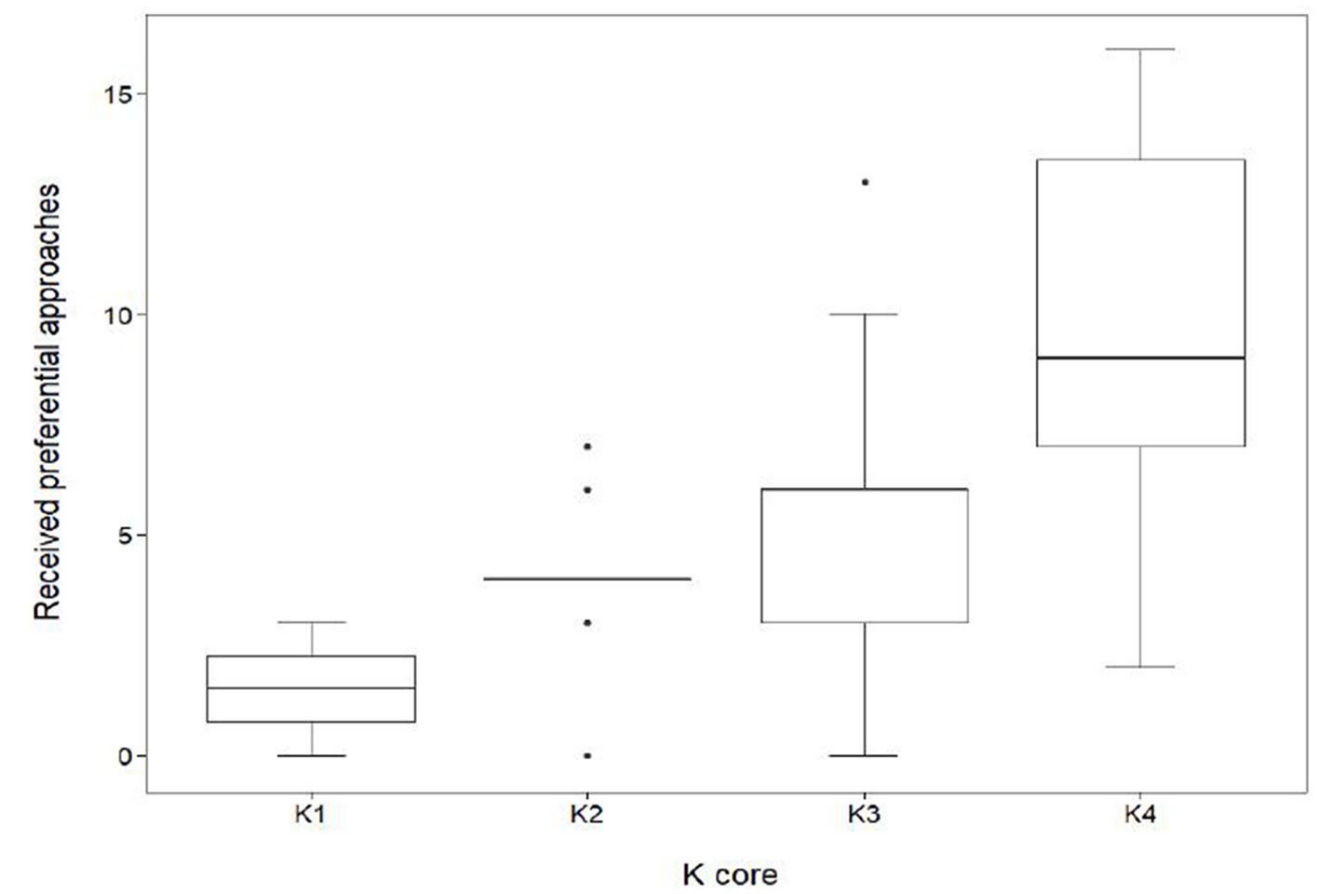
Distribution of received preferential associations between *k*-cores in the mean network (*n* = 70). In-degree centrality quartiles for the subgroups K1, K2, K3, and K4. Sows quantified in K1 (*n* = 4) had a median in-degree value of 1.5. The maximum K1 in-degree centrality was 3 with a minimum value of 0. Sows quantified in K2 (*n* = 10) had a median in-degree value of 4. The maximum K2 in-degree centrality was 7 with a minimum value of 0. Sows quantified in K3 (*n* = 29) had a median in-degree value of 6. The maximum in-degree centrality was 13 with a minimum value of 0. Sows quantified in K4 (*n* = 27) had a median in-degree value of 9. The maximum in-degree centrality was 16 with a minimum value of 2.

K-core had a significant effect on the number of received preferential associations (in-degree centrality) that occurred between the subgroups (Figure 5). Individuals within K4 received, on average, 9.7 ± 4.2 SD approaches, a value significantly higher (GLMM, coef. 0.58, z 4.4, *p* < 0.001) than those in K1 (1.5 ± 1.3SD), K2 (4.0 ± 1.8SD) and K3 (5.4 ± 3.1SD).

**Figure 6:**
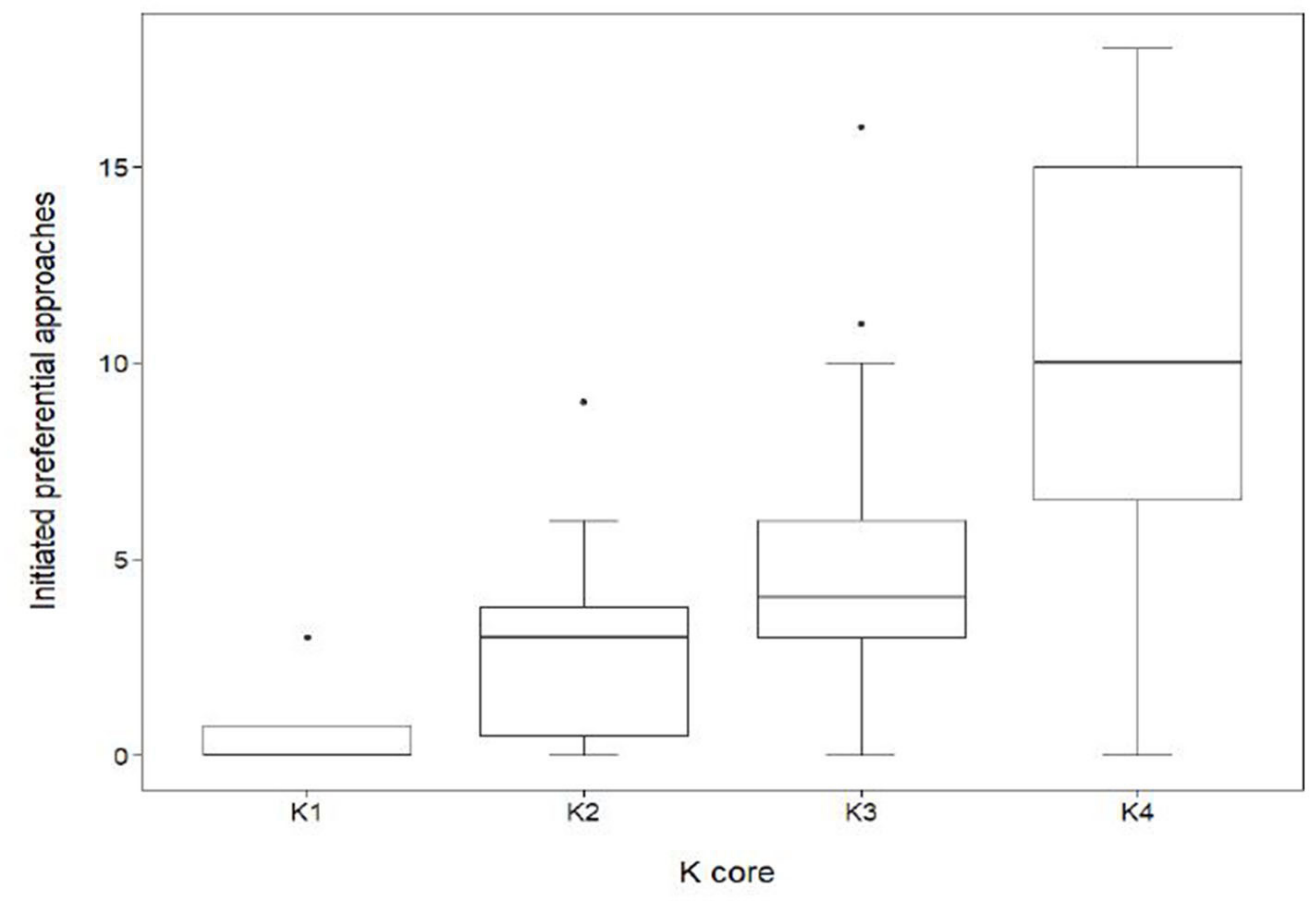
Distribution of initiated preferential associations between *k*-cores in the mean network (*n* = 70). Out-degree centrality quartiles for the subgroups K1, K2, K3, and K4. Sows quantified in K1 (*n* = 4) had a median out-degree value of 0. The maximum K1 out-degree centrality was 3 with a minimum value of 0, giving a range of 3. Sows quantified in K2 (*n* = 10) had a median out-degree value of 3. The maximum K2 out-degree centrality was 9 with a minimum value of 0. Sows quantified in K3 (*n* = 29) had a median out-degree value of 4. The maximum out-degree centrality was 16 with a minimum value of 0. Sows quantified in K4 (*n* = 27) had a median out-degree value of 10. The maximum out-degree centrality was 18 with a minimum value of 0.

The results also show that the sociability of an individual had a significant effect on connectedness within the herd (Figure 6). Sociability was measured by the frequency of initiated approaches made by an individual, including approaches to sows within other subgroups. Individuals within K4 initiated an average of 10.4 ± 5.1 SD initiated approaches, a value significantly higher (GLMM, coef. 0.72, z 4.2, *p* < 0.001) than those in K1 (0.8 ± 1.5 SD), K2 (3.0 ± 2.9 SD) and K3 (5.2 ± 3.5SD).

### 3.6. Sociability and parity

There were no significant differences between the frequency of initiated (out-degree centrality) or received (in-degree centrality) preferential associations and parity (Supplementary Table 2). The results indicate that in this study, parity was not a predictor of the sociability or popularity of an individual based upon a network of preferential associations.

### 3.7. Application of the brokerage typologies

**Figure 7:**
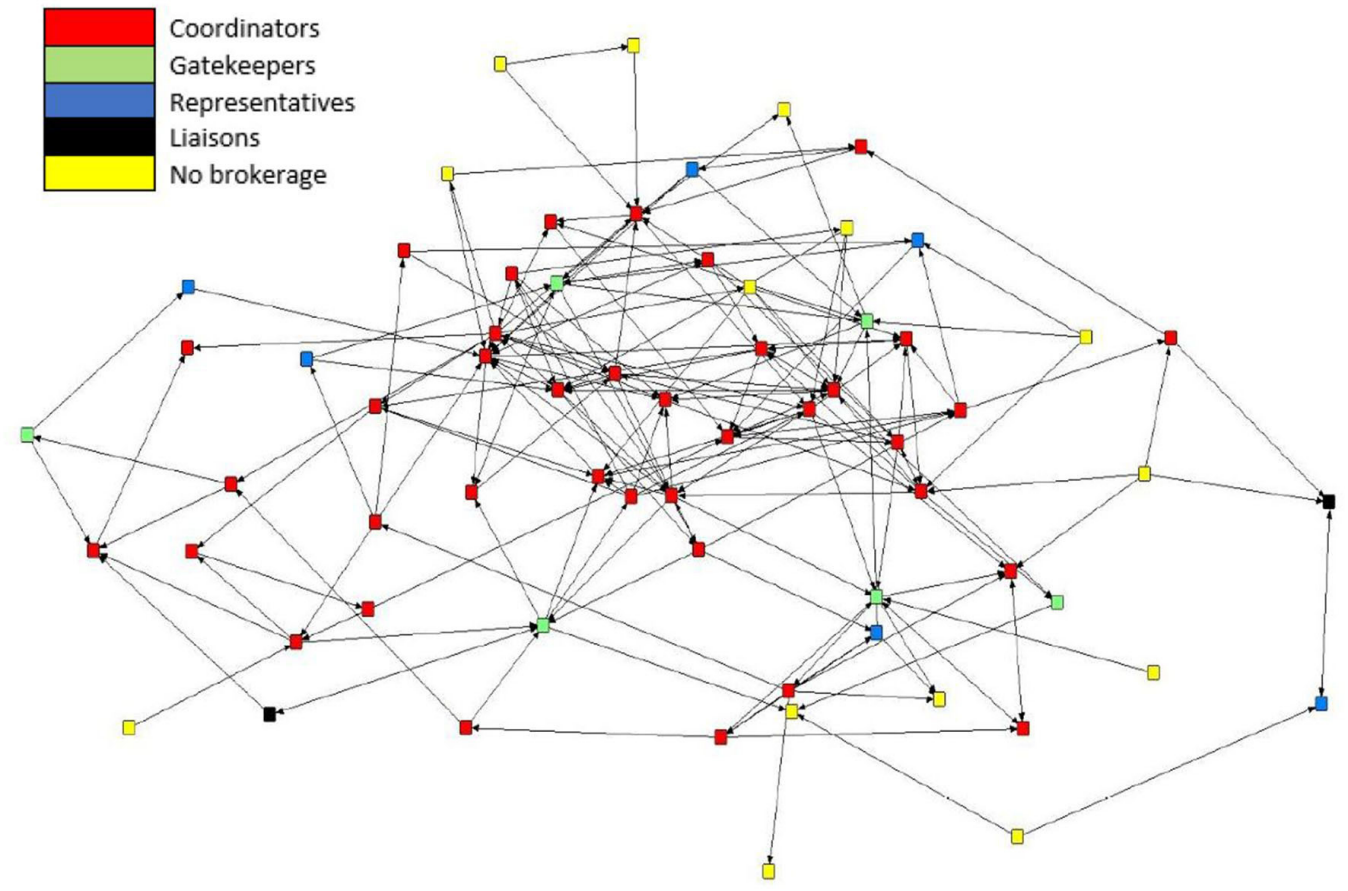
Directed sociogram for the mean preferential association network (n = 66) with brokerage typologies applied including coordinators (*n* = 38), gatekeepers (*n* = 6), representatives (*n* = 6), consultants (*n* = 0), liaisons (*n* = 2), and no brokering behavior (*n* = 14). The legend denotes the color codes for the brokerage types.

Brokerage typologies were applied to the subgroups of the mean preferential association network (*n* = 66). Due to inconsistencies in the normalized relative brokerage score, four sows were removed from the analysis, and these sows were removed from K3. The network consisted of four subgroups: K1 (*n* = 4); K2 (*n* = 10); K3 (*n* = 25); and K4 (*n* = 27). Within the herd, 79% (*n* = 52) of sows engaged in brokering behavior. Overall, the results show that the mean preferential association network was dominated by coordinators (Figure 7). The census count (Table 4) showed a higher propensity for engaging in coordinating behavior within the most highly connected sows (K4), with coordinating behavior representing 58% (*n* = 416) of all potential brokerage typologies. Coordinating behavior is described as brokering between unconnected sows of the same *k*-core. In comparison, sows in K3 predominantly engaged in gatekeeping behavior, described as brokering between unconnected sows, one within the same *k*-core as the gatekeeper and one belonging to a different *k*-core. Sows in K2 demonstrated the potential for the greatest reach within the network, with 50% of all brokering behavior revealed as liaising. Liaising is described as brokering between two unconnected sows in two different *k*-cores to which the liaison does not belong. Sows in the least connected subgroup (K1) did not engage in any of the five brokerage typologies.

**Table 4:** Census count of brokerage typologies within the *k*-cores (K1, K2, K3, and K4) in the mean preferential association network (*n* = 66).

***K-*core**	**Census count**	**Coordinator%**	**Gatekeeper%**	**Representative%**	**Consultant%**	**Liaison%**
K1 (*n* = 4)	0	0	0	0	0	0
K2 (*n* = 10)	6	0	17	33	0	50
K3 (*n* = 25)	89	44	31	25	0	0
K4 (*n* =27)	416	58	23	15	2	2

Brokerage typologies include coordinators, gatekeepers, representatives, consultants, and liaisons.

## 4. Discussion

Overall, the study herd demonstrated a general lack of cohesiveness, as indicated by the low-density results. As the threshold for preferential associations increased, the proportion of all possible dyadic ties decreased, with only 4 and 2% of potential connections made in the mean and 1.5 × mean networks, respectively. A lack of propensity to establish more sustained preferential associations as the threshold level increased is supported by the mean degree in each network. The sociograms of the original, mean, and 1.5 x mean networks showed a decline in cohesiveness, particularly in the 1.5 × mean network, in which the requirement to be included was ≥ 3 interactions between the same individuals. In this network, only 42 sows remained from the original network, consisting of nine components, of which six were dyads and only two reciprocated interactions. The greatest cohesion was observed in the original, unfiltered network (*n* = 78), a result of the inclusion of ties that occurred only once.

Single ties suggest randomness in the preferential selection, yet it is difficult to differentiate between extrinsic (e.g., the external factors including the physical and social environment) and intrinsic (e.g., the internal factors including inherent behaviors and individual differences) motivations for approaching or tolerating conspecifics, a confounder highlighted in previous related research [e.g., (3)]. Extrinsically, lying together is an essential function in social groups, particularly in pregnant sows, which have a lower critical temperature (14°C) due to feeding restrictions (64). However, as the threshold for interactions increased within the filtered networks, it was recorded that some individuals returned to the same conspecific up to five times. During the study, individuals had full access to the functional areas of the dry barn, including the straw-bedded resting area and the passageway in which the feeders and drinkers are located. With a variable stocking density in the dry barn and an ~2.32 m^2^ space allocation per pig, higher than the recommendation of the Red Tractor Assurance guidelines for pigs (65), opportunities for conspecific avoidance were available.

The lack of propensity to establish more sustained preferential associations as the threshold for inclusion increased was supported by the mean degree in each network (original = 12.91, mean = 5.1, 1.5 x mean = 0.88). The findings are consistent with previous research, which also documented positive behaviors as being rarer in commercial herds than in agonistic encounters (6). The reciprocity values of preferential ties were low throughout the study, with little variance between networks. The results imply that these preferential approaches were not mutually beneficial, despite the proximity of the initiator being tolerated, a suggestion supported by Voelkl (66) who proposed generalized reciprocity in unstructured networks. Furthermore, it is highlighted that reciprocity of behavior was not a motivator for repeatedly approaching the same individual.

The development of welfare and management approaches founded on mitigating detrimental experiences through positive behaviors in commercial species is an expanding area of animal behavior research (2, 67), so the question of why and with whom conspecifics bond is a pertinent one. Traditionally, prosocial behaviors are recognized as those that have positive effects at the individual and group levels, including caregiving, affiliation, sharing, social learning, and cooperation (2). However, differentiated behaviors such as preferential associations can have an alternative level of complexity when species are exposed to social changes to which they are not genetically predisposed, despite the benefits of engaging in such relationships. In complex, multilevel social groups, strong bidirectional ties (excluding mother-offspring ties) are shown to be beneficial to fitness and survival (5), including ties in unstable social groups (68), where demographic and social changes maintain population cohesion (69). Yet, the evolved social behavior of species adapted to the dynamics of a fission-fusion social structure does not reflect the natural behavior of the pig, shedding light on why individuals in the dynamic sow herd did not establish reciprocated strong bonds that could serve to mitigate the effects of a challenging environment.

The social context in an unstable commercial herd may have a more profound effect on the construction of ties and the extent to which their strength and reciprocity have fitness value at an individual level than what would be expected in a stable herd. Wild boars are predisposed to live in small, stable groups consisting of two to four genetically related mature sows (mothers and daughters or sisters), resulting in few but strong social bonds. These bonds are maintained through a cohesive, linear hierarchy in which conflict is reduced via the avoidance behavior of subordinates (70). Groups fracture when weaned piglets leave the herd to form their own separate groups (71). When wild boars are compared with feral domestic pigs, there is little variation in the expression of social behaviors (72), revealing that domestication has not suppressed the biological drive to form social groups or express behaviors that reflect those observed in their wild counterparts. Numerous factors impede the ability of intensively farmed pigs to construct and maintain such social ties, including large groupings, unfamiliar and unrelated conspecifics, and social instability caused by mixing. However, both wild and domestic pigs are behaviorally adaptive when presented with social or environmental challenges (73, 74).

Although accessing the strong social ties observed in wild and domestic feral individuals is difficult for intensively farmed pigs, their adaptive behavior may enable them to express their genetically predisposed behaviors through alternative social mechanisms. For example, in other species, a positive correlation between bonds and survival has been documented, as shown between Barbary macaques (*Macaca sylvanus*) and vervet monkeys (*Chlorocebus pygerythrus*). In these instances, the benefits of positive huddling behavior were observed in extreme weather conditions during the winter months (75, 76). In these examples, it is the number of bonds, not the quality of bonds, that is of value, and this is an important distinction and one that must be considered when evaluating the positive interactions between individuals in an unstable social environment. Not all species engage in strong preferential relationships to access fitness-enhancing benefits; the low quantity of reciprocated ties in the study herd is in line with recent research into the quality of strong bonds. Studies of female chacma baboons (*Papio ursinus*) show that although individuals who form some strong ties with conspecifics have fitness-enhancing effects, it is the development of weak ties that improves infant survival (77, 78).

Weak ties develop if there is little or no reciprocation in behavior, consistent with the low clustering coefficient seen in the herd. The clustering coefficient can determine whether triads of individuals are forming within a social group (56). If ties between individuals are strong (e.g., between A and B and between A and C), then strong ties are more likely to develop between other individuals within the triad (e.g., between B and C). In contrast, Granovetter (79) proposed that weak ties allow access to new information from various parts of a social network that would not be reached through the formation of strong ties and that strong ties can limit interactions with less familiar nodes within a network. Accordingly, the benefits of weak bonds are a significant motivator for tie strength development and sustainability. The existence of weak structural balance within the herd is consistent with the dynamic and unstable nature of the environment, where triadic ties are weak. Previous network studies have demonstrated that dynamic systems cause social imbalance due to a failure to engage in indirect “friendships,” in both ungulates and other mammals (80, 81). In this instance, it is the evolved behavior of a species that enables them to adapt their social strategies. For example, Sundaresan et al. (80) demonstrated different strategies to cope with fission-fusion societies and social instability in two closely related species of equids, through either the formation of close associations based upon sex and reproductive state or more generalized weak associations. The introduction of new sows to resident sows destabilizes the social structure, and the lack of reciprocated behavior reflects the strategy of forming weak associations, contravening their predisposition to form strong ties. Therefore, the lack of reciprocated behavior reflects a behavioral mechanism in the study herd that is potentially accessing some form of social support (in which reciprocation of behavior is not required) that is adapted and derived from the social context and physical environment.

This behavioral strategy was revealed when looking beyond the descriptive statistics. Initially, the metrics suggest that the study herd is not forming positive social relationships or engaging in social discrimination. When transitioning from the group to the individual level of analysis, the profile of behavior becomes intrinsically complex. In all three networks, centralization was low, indicating a level of social equality between conspecifics. When the *k*-cores (a measure of individual connectedness) were applied to the mean network, it showed the development of four subgroups (K1, K2, K3, and K4). Although the sows were not connecting at a high rate of interaction, individuals within one of the largest and most connected *k*-cores (K4, *n* = 27) were more socially central. Advanced analysis of the *k*-cores further revealed that sows in K4 were not just more social (initiating significantly higher levels of preferential approaches than conspecifics in other subgroups), but were also more popular, receiving significantly more approaches.

The application of the *k*-cores shows which pigs are more likely to engage in preferential associations (i.e., the most connected individuals), yet it does not show the contact patterns with whom they are interacting within and between subgroups. Sows in the most connected subgroups (K3 and K4) predominantly engaged in coordinating behavior, demonstrating preferential associations toward conspecifics of the same subgroup. Sows within the least connected subgroups (K1 and K2) did not engage in any coordinating behavior. The consistency of the relationship between brokerage type, connectedness, and behavior is in line with our previous findings. When brokerage typologies were applied to the aggression networks of dynamic sows in the same herd (49), a relationship between brokerage typology and behavior was also found, with sows in the most connected subgroups predominantly engaging in coordinating behavior and being the greatest initiators and recipients of aggression.

While the findings do not fully determine the development of mutual relationships, they reveal that preferential associations occur and, crucially, that these associations do not have to be bidirectional. There are individuals within the dynamic herd that have preferences for their social partner, regardless of a lack of reciprocation in behavior, suggesting a quality of social bonding not previously considered in commercial pigs. It is a finding supported by previous studies of allogrooming in dairy cattle, where asymmetric ties were more frequent than mutual ties (6, 43). The low clustering coefficient in the study herd (i.e., little triadic closure) is due to the lack of reciprocity.

In the absence of homophily by parity or familiarity, traits identified in other commercial species as motivators to form preferential ties (21, 53), alternative motivations for seeking asymmetric positive relationships must be considered. The most connected sows predominantly engaged in the highest rates of positive interactions through coordinating behavior, in which behavioral transmission is confined to conspecifics within their subgroup. In contrast, the lower-connected sows predominantly engaged in brokering behavior (gatekeeping, representing, and liaising) that interacted with individuals in other subgroups. These patterns of behavior may indicate a form of discrimination that is potentially driven by an individual's perceived sociality of a conspecific. The results showed that increased sociality (coreness value) raised an individual's social “profile” making them preferable in the networks compared with less connected sows. Kulahci and Quinn (82) recently proposed the concept of valuable social partners, and although it remains unclear what the benefits of associating with a highly connected sow may be, the concept suggests that valuable social partners provide a fitness-enhancing trait. In the absence of available benefits, such as access to foraging sites, the most likely motivation for a low-connected sow to approach higher connected sows at the study farm would be the preferred resting location. In this instance, an individual would not require reciprocation of behavior, just social tolerance, a response documented in poultry (83).

In contrast to lower-connected sows, the highest-connected individuals were found in the largest subgroups (K3 and K4), and unlike aggression, in which subgroup size may determine restrictions or increase interaction opportunities (84), motivations for interacting with conspecifics of the same subgroup (coordinating) may stem from an alternative source in positive behavior networks. One potential reason is “preferred competition,” which may explain the lack of reciprocation in behavior. Gutmann et al. (85) described preferential relationships as non-affiliative, competitive relationships between familiar animals where individuals prefer to interact both positively and negatively with familiar conspecifics. Although it cannot be assumed that sows within the same subgroup are familiar with each other due to the dynamics of the social environment, they may recognize an individual of equal social standing (based on connectedness), a strategy employed to support a degree of mutual social dominance in relationships. This explanation is supported by other studies that have identified correlations between preferential associations and agonistic interactions (23, 43).

## 5. Conclusion

The results show how individual variance in sociality at the group level has the potential to affect the social structure and cohesiveness of the group. Individual-level analysis reveals the complexities involved in decision-making when deciding with whom to engage in preferential associations. It remains unclear whether dynamic gestating sows form strong social bonds, yet the study reveals that positive ties are socially discriminatory, albeit asymmetric, and seemingly determined by perceived social standing within the herd. This presents opportunities to offer a measure of social support in an unstable social setting consisting of unfamiliar conspecifics. The novel measures of brokerage typologies highlight contact patterns determined by an individual's sociality, with the most connected sows predominantly engaging in coordinating behavior, offering enhanced insight into the social structure that extends traditional social network analysis methods. Similarities between brokerage type and connectedness in this study and our previous work on aggression networks highlight a pathway for future work to investigate the effects of social centrality in multiple behavioral networks on the welfare and production of dynamic sows.

## Data availability statement

The raw data supporting the conclusions of this article will be made available by the authors, without undue reservation.

## Ethics statement

The animal study was reviewed and approved by Writtle University College.

## Author contributions

SJ contributed to the conception, design, acquisition of data, statistical analysis, interpretation of the data, and writing of the first draft of the manuscript. SJ and JA contributed to revising the manuscript. All authors have read and approved the submitted version of the manuscript.
