# Supplementary figures and images for: Preferential associations in an unstable social network: applying social network analysis to a dynamic sow herd
Source: Front Vet Sci. 2023 Jun 1;10:1166632. doi: 10.3389/fvets.2023.1166632 (PMC10267343; doi:10.3389/fvets.2023.1166632)

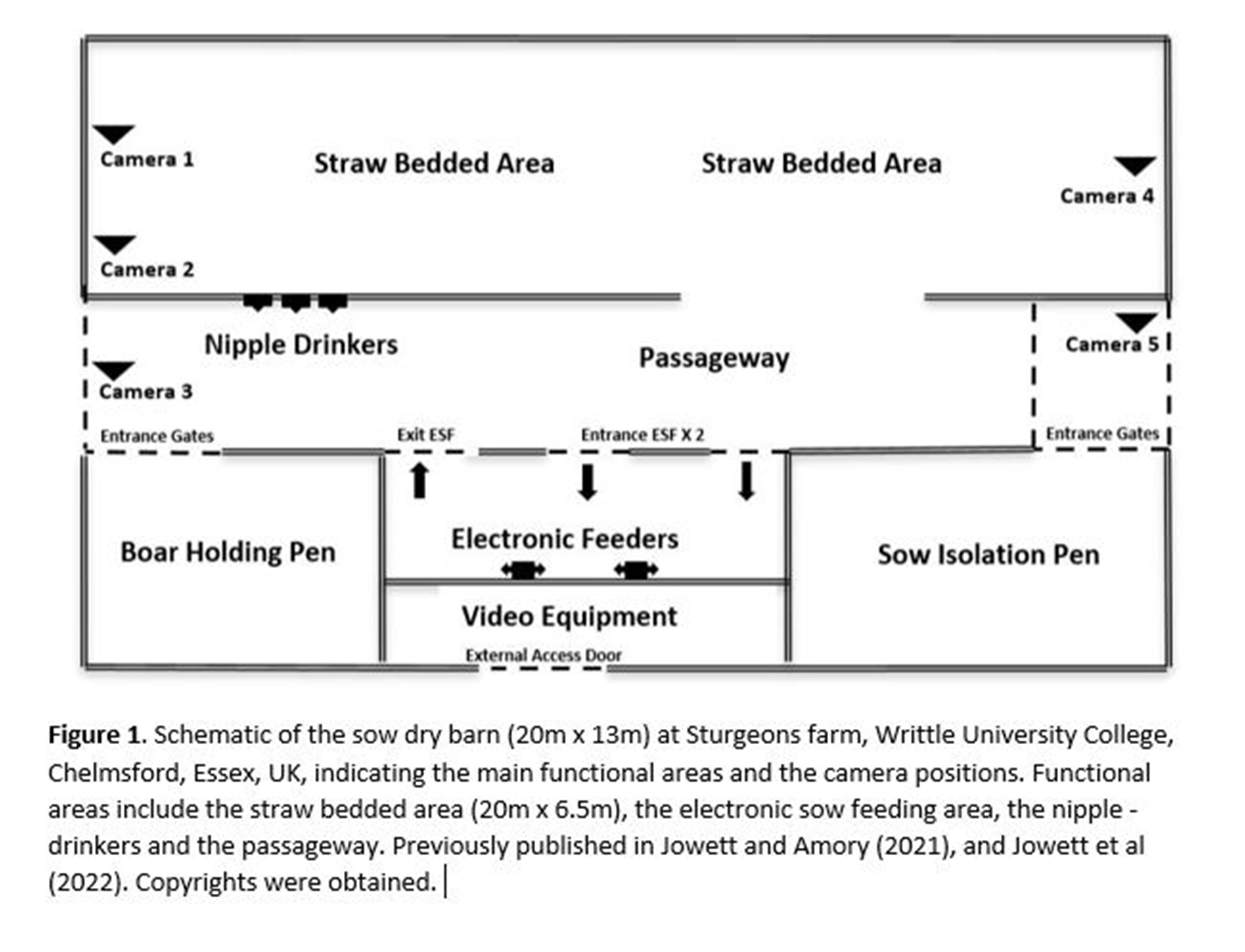

Supplement: Supplementary file 1 [file Image_1.jpg]

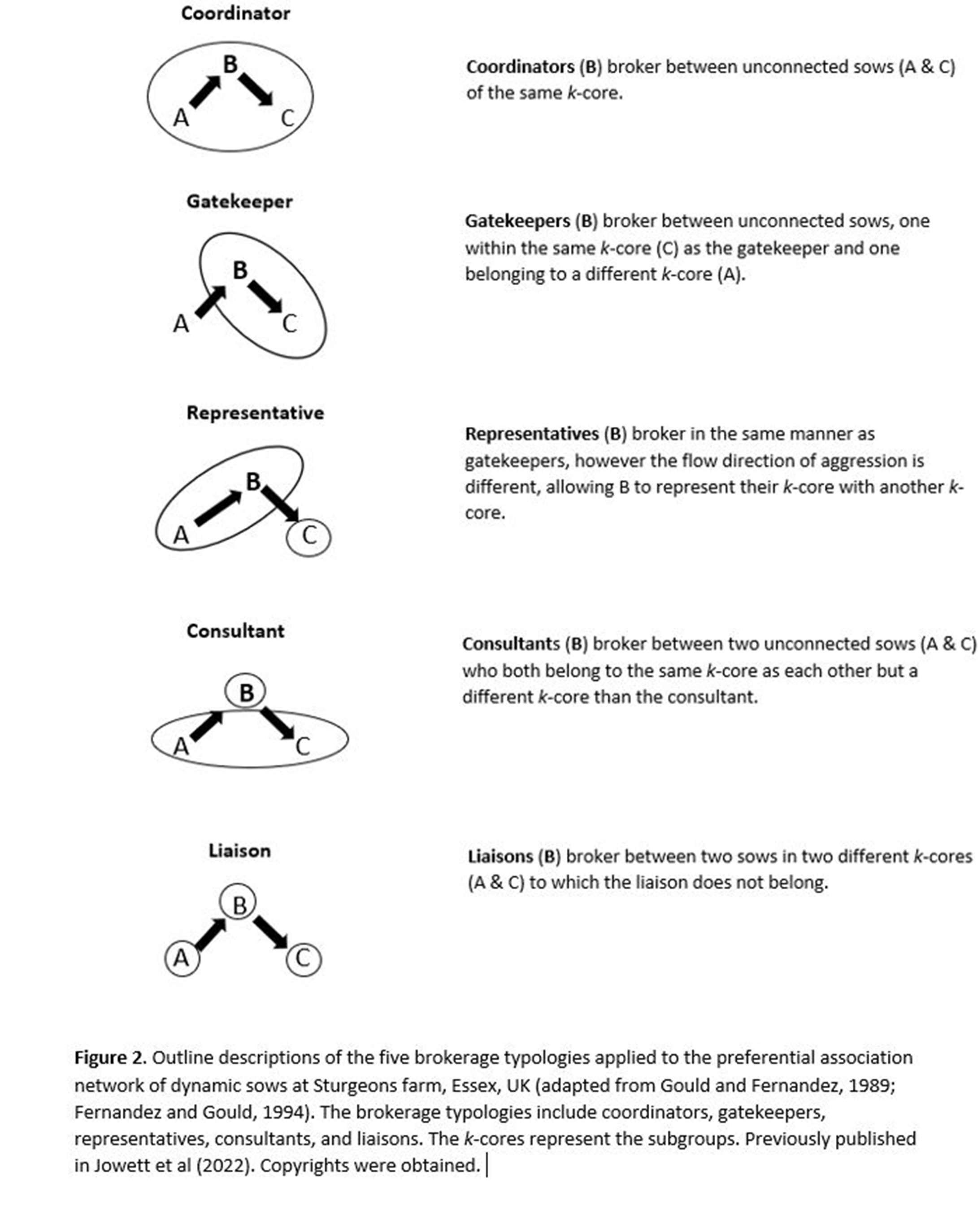

Supplement: Supplementary file 2 [file Image_2.jpg]
