# Supplementary material for: Preferential associations in an unstable social network: applying social network analysis to a dynamic sow herd
Source: Front Vet Sci. 2023 Jun 1;10:1166632. doi: 10.3389/fvets.2023.1166632 (PMC10267343; doi:10.3389/fvets.2023.1166632)
Supplement: Supplementary file 3 [file Table_1.docx]

**Supplementary Table 1**. External-Internal Index (E-I Index) applied to the mean preferential association network (*n* = 70). Ego-similarity for parity (1-6), breeding group (1-7) and k-core (K1, K2, K3, K4). Internal ties refer to ties with an individual of attribute similarity (homophily). External ties refer to ties with an individual of attribute difference (heterophily). Previously published in Jowett and Amory (2021). Copyrights were obtained.

|  | Internal ties | % Internal ties | External ties | % External ties | Expected E-I Index | E-I Index | Std error | |
| --- | --- | --- | --- | --- | --- | --- | --- | --- |
| **Parity** | 130 | 35.9 | 232 | 64.1 | 0.481 | 0.282 | 0.069 |  |
| **Breed Grp** | 118 | 32.6 | 244 | 67.4 | 0.696 | 0.348 | 0.051 |  |
| ***K*-core** | 248 | 68.5 | 114 | 31.5 | 0.331 | -0.370 | 0.070 |  |
