# Supplementary material for: Preferential associations in an unstable social network: applying social network analysis to a dynamic sow herd
Source: Front Vet Sci. 2023 Jun 1;10:1166632. doi: 10.3389/fvets.2023.1166632 (PMC10267343; doi:10.3389/fvets.2023.1166632)
Supplement: Supplementary file 4 [file Table_2.DOCX]

**Supplementary Table 2**. Mean indegree centrality (received preferential ties) and outdegree centrality (initiated preferential ties) by parity (1-6) in the preferential association network.

| Parity | Mean indegree centrality ± SD | Mean outdegree centrality ± SD |
| --- | --- | --- |
| 1 | 6.4 ± 4.47 | 6.4 ± 3.34 |
| 2 | 6.4 ± 3.75 | 7.0 ± 6.00 |
| 3 | 7.2 ± 4.41 | 7.1 ± 5.09 |
| 4 | 4.8 ± 3.04 | 5.3 ± 5.53 |
| 5 | 7.3 ± 5.13 | 5.7 ± 2.52 |
| 6 | 8.4 ± 6.35 | 7.0 ± 7.42 |
